# Single-Time Gastroscopy in High-Risk Patients: Screening Effectiveness for Gastric Precancerous Conditions in a Low-To Moderate-Incidence Population

**DOI:** 10.3390/jcm14196910

**Published:** 2025-09-29

**Authors:** Krystian Ciechański, Erwin Ciechański, Krystyna Kłosowska-Kapica, Barbara Skrzydło-Radomańska

**Affiliations:** 1 Department of Gastroenterology, Regional Specialist Hospital, 20-781 Lublin, Poland; 2 Department of Toxicology, Medical University of Lublin, 20-090 Lublin, Poland; 3 Department of Cardiology, 1st Military Clinical Hospital with the Outpatient Clinic, 20-049 Lublin, Poland; 4 Department of Gastroenterology with Endoscopy Unit, Medical University of Lublin, 20-954 Lublin, Poland

**Keywords:** gastric cancer, gastric precancerous conditions, atrophic gastritis, intestinal metaplasia, dysplasia

## Abstract

**Background:** Gastric cancer (GC) is the fifth most common malignancy worldwide. Early detection of precancerous conditions—atrophic gastritis (AG), intestinal metaplasia (IM), and dysplasia—is vital for surveillance. **Objectives:** To assess the accuracy of single high-quality endoscopy (HQE) in detecting advanced GPCs and to identify risk factors for AG, IM, and dysplasia. **Methods:** A retrospective review of 442 gastroscopies (2017–2022) at a single center. Endoscopic findings were compared with histology, including OLGA/OLGIM staging, dysplasia, and *Helicobacter pylori* (*H. pylori*) status. **Results:** The study population comprised 319 women (72.17%) and 123 men (27.83%), with a mean age of 59 years (SD: 12.53). AG, as defined by OLGA and OLGIM staging, was identified in 90 patients (20.36%) and 50 patients (11.31%), respectively. A total of 44 cases of de novo gastric dysplasia were observed, while *HP* infection was confirmed in 37 individuals (8.37%). We observed similar low sensitivity for detection of advanced OLGA (32.5%), OLGIM (40%), and dysplasia (19.7%) with relatively high specificity (~89%). Advanced AG and IM peaked at ages 51–53. Risk factors for advanced OLGIM included male sex (OR 2.26; *p* < 0.001) and presence of dysplasia (OR 2.09; *p* = 0.02). Dysplasia was positively associated with AG (OR 2.03; *p* < 0.001) and IM (OR 2.21; *p* < 0.001) but inversely associated with a family history of GC (OR 0.44; *p* < 0.001). **Conclusions:** A single HQE can help exclude advanced GPCs, but due to low sensitivity, gastric mapping biopsies remain crucial. Males are at increased risk of extensive IM. Family history of GC was linked to lower OLGA/OLGIM stages.

**Simple Summary**
Early detection of precancerous conditions (GPC) including atrophic gastritis (AG), intestinal metaplasia (IM), and dysplasia is crucial for gastric cancer (GC) management. This retrospective study analyzed 442 single gastroscopies to evaluate the relationship between endoscopy and histological findings including Operative Link for Gastritis Assessment (OLGA) and Operative Link for Gastric Intestinal Metaplasia Assessment (OLGIM) stagings in detecting advanced gastric precancerous conditions (GPCs) in patients with GC risk factors. Despite high specificity (~89%), endoscopic sensitivity was low (32.5% for OLGA III/IV, 40% for OLGIM III/IV), highlighting the need for systematic gastric mapping biopsies. Male sex and dysplasia were linked to a higher risk of extensive gastric IM. Interestingly, patients with a family history of gastric cancer more often had OLGA/OLGIM 0-II. These findings emphasize the importance of personalized risk assessment, integrating demographic and environmental factors in planning optimal time of screening gastroscopy and surveillance in high-risk GC populations.
**What’s New**
Our study is one of the few that assesses the effectiveness of a single high-quality gastroscopy in identifying precancerous gastric conditions in a low-to-moderate-incidence population (Poland). We observed that individuals with a family history of stomach cancer more often had lower OLGA/OLGIM stages, which may indicate earlier detection of changes and the effectiveness of previous H. pylori eradication. Furthermore, we demonstrated that male sex is associated with a higher risk of advanced intestinal metaplasia, highlighting the need for individualized surveillance strategies. Despite the use of a high-quality endoscopic protocol, diagnostic sensitivity remains low, confirming the importance of systematic mapping biopsies in this patient group and the need for further training of endoscopists.

## 1. Introduction

**Figure 1:**
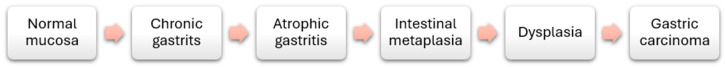
The gastric precancerous cascade.

Gastric cancer (GC) poses as important worldwide challenge, being classified by the WHO as the fifth most common cancer globally [1]. Esophagogastroduodenoscopy (EGD) with biopsies persist as standard procedure for detecting GC and gastric precancerous conditions (GPC). For the development of GC, several risk factors have been identified, including family history, environmental habits (diet, alcohol consumption, smoking), autoimmune or genetic disorders, and infection with *Helicobacter pylori* (*H. pylori*) which is the most important risk factor [2]. The development of gastric cancer is often preceded by gastric precancerous conditions (GPC) including atrophic gastritis (AG), intestinal metaplasia (IM), and dysplasia [3], Figure 1. The presence of high-stages (stages III-IV) of Operative Link for Gastritis Assessment (OLGA) or Operative Link for Gastric Intestinal Metaplasia Assessment (OLGIM) are also correlated with the increased risk of gastric cancer [4,5]. Once identified, these conditions may indicate the need for initiating surveillance programs aimed at monitoring patients more closely for dysplasia or GC and providing appropriate treatment if indicated [6]. Current preventive strategies for GC include *H. pylori* eradication and histological risk assessment to identify candidates for such surveillance. Although population-based screening is not recommended in low-risk countries, such as Poland (ASR 8.1/100.000 person-years), endoscopic surveillance in selected high-risk groups may offer benefits [6,7]. Besides white-light endoscopy (WLE), new evidence indicates that access to techniques including virtual chromoendoscopy (VCE), specific training in GPC detection, proper time of EGD or cleanliness of mucosa improve detection of GPC and GC [8,9,10].

The aim of this study was to retrospectively assess the effectiveness of single-time high-quality EGD in identifying advanced GPC in patients with known GC risk factors. Special attention was paid to identify risk factors for GPCs in relationship with gastroscopy indication, sex or age. Secondary goal was to correlate our results with selected risk factors and indication for endoscopy and advanced OLGA/OLGIM or dysplasia for better understanding and planning targeted surveillance strategies.

## 2. Material and Methods

We conducted a single-center, retrospective study over a five-year period. High quality single-time EGD was performed either as a follow-up examination or as a screening procedure in patients with a family history of gastric cancer as single indication. A family history of GC was defined as a presence of GC in first-degree relatives.

Patients were eligible if they met at least one of the following:

1. Family history of gastric cancer (GC) and either:
  - Age ≥45 years and no prior gastroscopy or
  - Last gastroscopy performed ≥3 years ago.
2. Extensive gastric intestinal metaplasia (IM), (OLGIM III/IV) documented in previous reports, with last gastroscopy performed ≥3 years ago.
3. Extensive gastric atrophy (AG), (OLGA III/IV) documented in previous reports, with last gastroscopy performed ≥3 years ago.
4. Gastric dysplasia (low grade/indefinite) documented in previous reports, with last gastroscopy performed ≥1 year ago.

The chosen endoscopy intervals (≥3 years for AG/IM, ≥1 year for LGD) were based on current MAPS III recommendations and aimed to capture meaningful progression without including patients too soon after prior assessment. The inclusion criteria for individuals with family history of GC (age ≥45 years and no prior endoscopy, or prior endoscopy ≥3 years ago) reflect a new MAPS III approach of screening in 1st degree relatives of patients with GC.

**Figure 2:**
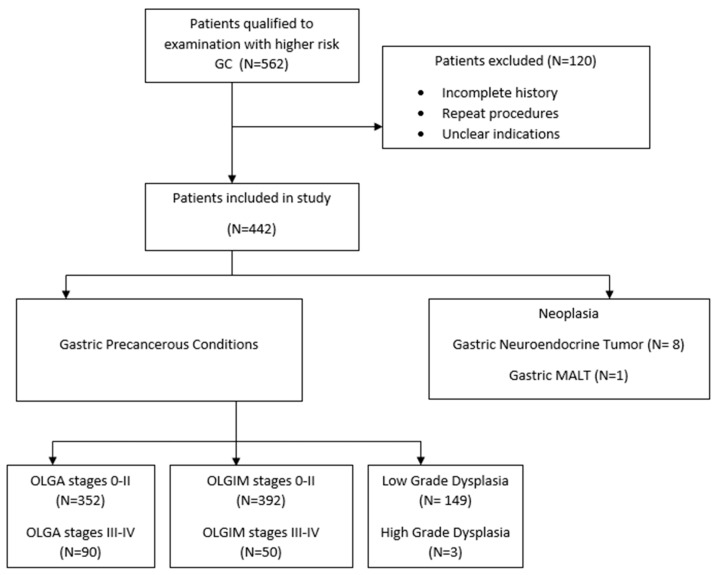
Flowchart.

Between June 2017 and July 2022, a total of 562 gastroscopies were performed. A total of 120 patients (21%) were excluded due to incomplete data, repeated procedures in the same individual or lack of clearly defined indications meeting inclusion criteria as shown in Figure 2.

Thus, 442 examinations were included in the analysis. Each endoscopic report contained patients’ personal data, sex, age, indication for endoscopy, endoscopic findings, and operator details. To standardize and enhance procedural quality, a dedicated protocol was implemented before beginning of the gastroscopic evaluation (2017).

High-Quality EGD Protocol:

1. Use of high-definition resolution Olympus^®^ (Hamburg, Germany) endoscopes with VCE, water jet and CO_2_ insufflation, no magnification nor distal cap.
2. Oral administration of 600 mg N-acetylcysteine solution 15 min prior to the examination to improve mucosal visibility [11].
3. Procedures performed under analgosedation, with intravenous midazolam and fentanyl.
4. Minimum procedure time of 15 min, with at least 7 min focused on gastric mucosa assessment using WLE and VCE.
5. Random biopsy sampling conducted in accordance with the updated Sydney protocol, with additional target sampling from suspicious areas [12].
6. Photo documentation comprising at least 22 images per procedure, following the systematic screening protocol (SSS), plus images of any abnormal findings [13].

All endoscopic procedures were performed by three experienced gastroenterologists with over >1000 EGD annually and at least 100 VCE annually. Biopsies were collected according to updated Sydney Protocol—two from antrum and one form incisura in one vial; two from corpus in another—to evaluate gastritis (OLGA/OLGIM staging) and *H. pylori* status (H&E staining and immunohistochemical labeling). Additional findings were sampled separately. All specimens were fixed in formaldehyde and transported under standard conditions to a single collaborating histopathologist, who assessed the samples with access to the full endoscopic report.

Macroscopic endoscopic findings were categorized as normal or suspected advanced gastric precancerous conditions (GPC), including the following: extensive gastric intestinal metaplasia (IM) or atrophy (AG), dysplasia, early cancer or suspicious unclassified lesions. Histological examinations provided definitive assessment of IM, AG, and dysplasia. OLGA and OLGIM stages were calculated from pathology reports [14]. Stages 0–II were classified as low-risk stage, while OLGA/OLGIM stages III and IV were classified as high-risk gastritis [6]. Dysplasia was categorized as low-grade dysplasia (LGD) or high-grade dysplasia (HGD) [3]. Endoscopic sensitivity and specificity for detecting advanced OLGA/OLGIM stages and dysplasia were assessed. Additionally, risk factors for OLGA/OLGIM stage III–IV or dysplasia were analyzed using univariate and multivariable logistic regression, adjusting for age, sex, and endoscopy indication. We selected variables for regression analysis based on their clinical relevance and the previous literature on gastric cancer.

## 3. Statistical Analysis

Qualitative features were presented as frequencies and percentages, while quantitative features were characterized by mean values and SD. For nonparametric comparisons, median and IQR were utilized. Multivariable logistic regression models were employed to assess the impact of selected risk factors such as age, sex, and indications for endoscopy on the odds of developing advanced stages of AG. Results were reported as OR with 95% CI. For statistical analysis, Pearson’s χ^2^ test was used to evaluate categorical variables. The efficiency of endoscopic diagnosis of GPC was reported as sensitivity, specificity, and positive/negative predictive value with visualizations provided by ROC curves. To assess whether the OLGA or OLGIM staging systems correlate with patient age, we analyzed the distribution of two age groups, using 45 years as the threshold. A *p* value below 0.05 was considered significant. The collected data were grouped on Microsoft Excel spreadsheets and analyzed with STATISTICA v.13.1 (StatSoft Inc., Tulsa, OK, USA).

## 4. Results

In this study, a total of 319 women (72.17%) and 123 men (27.83%) were examined, with a mean age of 59 years (SD;12.53). The prevalence of advanced gastritis, classified as OLGA III/IV and OLGIM III/IV, was found to be 90 cases (20.36%) and 50 cases (11.31%), respectively. *H. pylori* infection was confirmed in 37 examinees (8.37%). A total of 44 de novo gastric dysplasia (41 LGD and 3 HGD) were diagnosed, compared to patients’ indications. The prevalence of dysplasia was 34.39%. The predominant indication for endoscopy was a history of atrophic gastritis, present in 249 patients (56.33%). Demographic, endoscopic, and histological characteristics are detailed in Table 1.

Among the evaluated group, eight (1.81%) gastroenteropancreatic neuroendocrine tumors (GEP NETs), four (0.9%) early gastric cancers, three (0.68%) HGD, and one (0.23%) gastric MALT were diagnosed. Mean age (median) in OLGA 0–II and III–IV was 60.25 (57) and 52.44 (52.5), further in OLGIM 0–II, 59.61 (57) and III–IV, 51.22 (51) years, respectively.

Data comparing low- and high-risk stages of OLGA and OLGIM based on various factors are presented in Table 2. Endoscopic detection of OLGA III/IV showed a sensitivity of 32.50% and specificity of 89.77%, with a positive predictive value (PPV) of 41.93% and negative predictive value (NPV) of 83.15%. For OLGIM III/IV, sensitivity was 40.0%, specificity 89.29%, PPV 32.25%, and NPV 92.10%.

**Figure 3:**
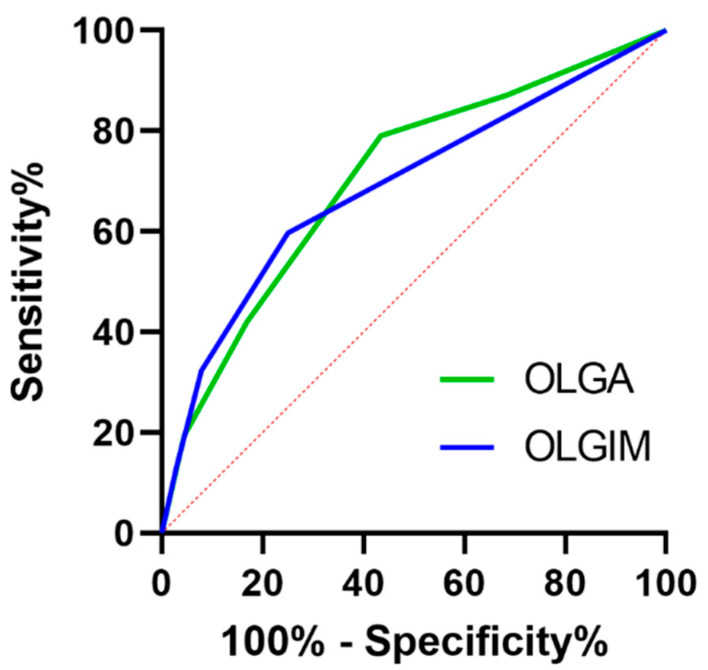
The association between endoscopy results in sensitivity and specificity in OLGA/OLGIM occurrence presented as receiver operating characteristic (ROC) curves with 95% confidence intervals. The green line represents the operative link on gastritis assessment (OLGA 0-IV) with area under the curve (AUC) of 0.76; blue line represents the operative link on gastric intestinal metaplasia (OLGIM 0-IV) with AUC of 0.77.

To compare sensitivity and specificity between the endoscopic findings and the OLGA/OLGIM stages, we conducted receiver operating characteristic (ROC) curve analysis, as illustrated in Figure 3.

The area under curve (AUC) of the ROC curve for OLGA III/IV was 0.7610 (0.0225), (95% Cl; 0.7169–0.8052) and for OLGIM III/IV, 0.7730 (0.0220) (95% Cl; 0.7299–0.8162), with both *p* < 0.001.

The presence of a family history of gastric cancer was significantly correlated with the development of lower-grade OLGA (0–II) and OLGIM (0–II) compared to OLGA III-IV (42.36% vs. 14.44%; *p* < 0.001) and OLGIM III-IV (39.53% vs. 14.00%; *p* < 0.001). No correlation was found between sex and OLGA stages; however, male sex was significantly associated with all OLGIM stages (*p* < 0.001). Endoscopy results showed that in the vast majority in the normal endoscopy group, low stage OLGA/OLGIM were present. Moreover, in the suspicious group, low-stage OLGA/OLGIM occurred in 36 (58%) and 42 (67%) cases as in comparison with high-grade OLGA/OLGIM in 26 (43%) and 20 (32%) cases, respectively. Finally, a significant correlation was observed between endoscopic mucosal appearance (normal/suspicious) and low-risk versus advanced OLGA/OLGIM stages, respectively (*p* < 0.001).

Using 45 years as the threshold, we analyzed OLGA/OLGIM severity by age Table 3.

Among patients ≥45 years (*n* = 315), 15% had OLGA III–IV, while in those <45 years (*n* = 127), 32.28% had OLGA III–IV. A significant correlation was found between older age and OLGA/OLGIM 0-II stages (*p* < 0.001).

We also evaluated patients to assess the risk of developing high-stage OLGA III-IV/OLGIM III-IV in relation to sex and dysplasia, as shown in Table 4.

Univariate logistic regression revealed that male sex (OR 2.26; 95% CI: 1.24–4.14; *p* = 0.001) and presence of dysplasia (OR 2.09; 95% CI: 1.13–3.88; *p* = 0.02) were significantly associated with advanced OLGIM. Finally, multivariable logistic regression analysis revealed that the risk of OLGA III-IV is increased in patients with AG (*p* < 0.001; OR, 6.48; 95% CI, 3.22–13.03;) Table S1 Supplementary Material.

Among 380 normal mucosal status endoscopies, dysplasia was detected in 122 cases (32%), and in 62 suspicious cases, 30 (48%) had dysplasia. There was significant correlation between suspicious endoscopy image and presence of dysplasia (*p* = 0.01). Endoscopic sensitivity for detecting dysplasia was 19.74%, specificity 88.97%, PPV 48.39%, and NPV 67.89% Table S2 Supplementary Material.

As shown in Table 5, univariate models identified several factors associated with increased dysplasia risk: history of gastric atrophy (OR 2.03; 95% CI: 1.34–3.05; *p* < 0.001), history of intestinal metaplasia (OR 2.21; 95% CI: 1.47–3.32; *p* < 0.001), and family history of GC were inversely associated (OR 0.44; 95% CI: 0.28–0.67; *p* < 0.001). Multivariable regression identified history of IM as an independent predictor (OR 1.72; 95% CI: 1.08–2.73; *p* = 0.02). Sex and age were not significant Table S3 Supplementary Material.

## 5. Discussion

According to global prognosis, the incidence and morbidity of GC remain at high levels and is expected to have a rising trend among younger patients, resulting in a current significant public health challenge worldwide [15]. In Poland, the incidence of GC is 5.0 per 100,000 in women and 12.2 per 100 000 in men, classifying the Polish male population as intermediate-risk for developing GC [7].

*H. pylori* status according to WHO remains as the leading risk factor for GC worldwide [16]. Its successful eradication has been proven to cure gastric mucosal inflammation and reverse gastric atrophy if intestinal metaplasia is not present, preventing its progression to GC [17]. The relatively small numbers of detected *H. pylori* infections (8.37%) in our study for such burdened cohort probably resulted from the fact that most of the patients had previously undergone an EGD or non-invasive test with *H. pylori* screening with eradication if present. Numerous cases of gastric IM and advanced GPC, with a relatively small number of active *H. pylori* infections, indicate previous successful management. On the other hand, *H. pylori* reinfections or unsuccessful treatment, could have contributed to the increased detection of dysplasia in our cohort. However, the retrospective nature of the study and lack of follow-up limited assessment of regression in gastric atrophy post-eradication. Moreover, previous *H. pylori* infection status was not included in the inclusion criteria; its presence was only validated in histology reports.

Our results showed high specificity and negative predictive values in endoscopic diagnosis of both OLGA/OLGIM III/IV. Analyzing specificity in endoscopic findings of advanced stages of OLGA and OLGIM, our results were similar with other studies, positioning at high levels with 89.77% and 89.29%, respectively [18,19]. However, sensitivity for detecting advanced atrophic gastritis (AG) and IM using VCE was low (32.5% and 40%). This aligns with the literature indicating high specificity but low sensitivity for endoscopic IM diagnosis [20]. While magnifying VCE can improve sensitivity to 80–85% according to meta-analyses, our findings were only slightly better than earlier Polish data and showed no significant improvement with normal VCE [19,21]. Additionally, AI-assisted systems for detecting gastric precancerous conditions (GPC) show promising potential [22].

Despite high specificity and NPV, the diagnostic sensitivity and PPV for AG and IM remain low in routine clinical practice, as well as in other Western studies [19], supporting the need for gastric mapping in patients with suspected GPC and proper training.

According to MAPS III guidelines, patients with LGD (or indefinite) with no lesions seen during endoscopy should be referred for high-quality endoscopy. If no lesions are found again, endoscopic surveillance should last 12 months [6]. In our study, gastric dysplasia was identified in 34.39% of patients, including 108 individuals with a history of low-grade dysplasia. In histological assessment, a total of 152 cases of LGD and HGD were found. However, cases with incomplete dysplasia were summarized together with LGD, due to the surveillance need. Thus, level of dysplasia detection may be falsely increased.

Our study showed high specificity (88.97%) but low sensitivity (19.74%) for detecting dysplasia. This may suggest that many dysplastic lesions found previously were not visible during our examination; thus, biopsies were taken from areas suspected for advanced AG/IM rather than dysplasia. Despite the low sensitivity, our study led to 44 de novo diagnoses of gastric dysplasia during a single endoscopy, which seems to be a substantial number for such a cohort. These results may suggest the need for more frequent surveillance in this group of patients, or improvement in the quality of the endoscopy for better visualization of dysplastic lesions.

Univariate logistic regression revealed a significantly increased risk of dysplasia in patients with a history of IM (OR 2.21), or AG (OR 2.03) on previous endoscopies regardless extensive of lesions. Interestingly, a family history of gastric cancer was associated with decreased dysplasia risk (OR 0.44). This may be due to greater health awareness in these individuals, more frequent endoscopic screening, prior *H. pylori* eradication and healthier lifestyle choices such as reduced alcohol consumption and smoking cessation.

The predominance of women in the study (72.17%) reflects real trends in referral for EGD, as women are more likely to participate in preventive examinations, particularly in the presence of family history or prior histological changes. All analyses were adjusted for sex; no significant association was found between sex, advanced OLGA stages, and dysplasia, whereas male sex emerged with a more than twofold increased risk for advanced OLGIM stages in univariate logistic regression. When comparing both genders, we found that prevalence of IM was significantly higher in men. Therefore, this group may warrant closer clinical attention, potentially through more frequent surveillance or even targeted screening, particularly when additional risk factors are present.

A family history of GC is a well-known risk factor of gastric cancer, typically associated with a two- to threefold increase in gastric cancer. In the Chinese study, Min Wu et al. proved that family history of GC was significantly associated with OLGA stages III–IV [23]. However, in contrast to Poland, this country is a high-risk GC incidence region. In our study, patients with family history of GC, significantly, often presented low-risk OLGA/OLGIM (0–II); moreover, we observed results that were the opposite of what we expected, with lower risk of developing OLGA/OLGIM III–IV. This finding may reflect earlier detection of GPCs and more effective clinical management with this group of patients, including *H. pylori* eradication and lifestyle modifications, which likely contribute to the inhibition of IM or AG progression.

In our study, the proportion of patients with advanced OLGA/OLGIM stages was significantly higher in those under 45 years, with a significant inverse correlation between age and OLGA/OLGIM stage. Although this appears contradictory to previous reports, our cohort mainly included patients undergoing follow-up endoscopy, (except those with a family history of GC as single factor).

Based on current MAPS III and Maastricht VI/Florence guidelines, screening gastroscopy with risk stratification of precancerous lesions is recommended for patients aged ≥ 45 years with a positive family history of GC [6,17]. In contrast, the British Society of Gastroenterology recommends screening in individuals aged ≥ 50 with high-risk features, including pernicious anemia, male sex, smoking and/or a positive family history of GC [24]. Polish studies have explored optimal age for single gastroscopy in detecting esophageal and gastric precancerous conditions, by analyzing benefit-to-harm ratio and potential acceptable patients’ age with range for such screening between 40 and 69 years [25,26]. While our group did not show strong age-related trends in advanced GPCs, we found that men more often had advanced gastric IM. Mean ages for advanced AG, IM, and dysplasia were 52.4, 51.2, and 56.3 years, respectively. These findings are promising but require validation in larger studies.

## 6. Limitations

The study was conducted in a single center, without long-term follow-up. Three different operators performed the endoscopies and tissue collections, introducing potential outcomes variability. The pathologist had access to endoscopic results, which may have influenced the histological reports. We were unable to fully validate the indications for endoscopy, including the extent of IM/AG or family history relativeness of GC. Screening and surveillance patients were analyzed together, which increases sample size, includes all clinically relevant subgroups, and reflects a real-world scenario, but may represent a potential source of bias. Because of the retrospective design and the study period preceding MAPS III, EGGIM classification could not be applied, and suspicious lesions were reported in an aggregated form, which limits lesion-specific accuracy assessment. Some patients had more than one indication for gastroscopy, which may have affected our analysis outcomes. Additionally, the endoscopic diagnostic sensitivity for IM, AG, and dysplasia may have been underestimated due to the classification of lesions suspected for gastric precancerous conditions (GPC). *H. pylori* history status was not taken into consideration in the inclusion criteria. Incomplete dysplasia was summarized together with LGD.

## 7. Future Directions

Future studies should validate these findings in larger, prospective, multicenter cohorts, with clear separation of screening and surveillance populations. Incorporating detailed *H. pylori* history, lifestyle factors, and medication use will improve risk stratification. The utility of advanced endoscopic and AI-assisted techniques in real-world settings, along with cost-effectiveness analyses of surveillance strategies in intermediate-risk populations, also warrants further investigation. Finally, sex-specific and age-specific risk profiles should be explored to refine tailored screening and surveillance algorithms.

## 8. Conclusions

A single high-quality gastroscopy demonstrates strong clinical utility in excluding advanced GPCs. However, due to its limited diagnostic sensitivity in a real-life clinical setting, risk stratification through systematic gastric mapping biopsies remains essential.

Patients with a family history of gastric cancer more often presented with lower OLGA/OLGIM stages and had a lower risk of advanced lesions, which may reflect the effectiveness of the *H. pylori* “test and treat” strategy.

Male sex was associated with an increased risk of extensive gastric IM, highlighting the influence of sex-related and possibly environmental risk factors.

These findings underscore the importance of individualized gastric cancer risk assessment, including demographic and environmental factors, to determine screening and surveillance strategies.

## Figures and Tables

**Table 1:** Endoscopic, histologic, and metrical characteristics of study population. Abbreviations: The Interquartile Range (IQR), Operative Link for Gastritis Assessment (OLGA), Operative Link for Gastric Intestinal Metaplasia Assessment (OLGIM), Low-grade dysplasia (LGD), High-grade dysplasia (HGD), Gastroenteropancreatic neuroendocrine tumors (GEP NETs), Gastric mucosa-associated lymphoid tissue (MALT).

Parameter	Value
Patients, *n*	442
Age	Years
Mean (SD)	59 (12.53)
Median (IQR)	56 (16)
Minimum	33
Maximum	93
Sex	
Men/Women, *n*	123/319
Indications for endoscopy, *n* (%)	
Family history of gastric cancer	160 (36.2)
History of atrophic gastritis	249 (56.33)
History of intestinal metaplasia	226 (51.13)
History of dysplasia	108 (24.43)
Endoscopic results, *n* (%)	
Normal	380 (85.97)
Suspicious	62 (14.03)
Histological results, *n* (%)	
OLGA 0–II	352 (79.64)
OLGA III–IV	90 (20.36)
OLGIM 0–II	392 (88.69)
OLGIM III–IV	50 (11.31)
Dysplasia LGD	149 (33.71)
Dysplasia HGD	3 (0.68)
Additional findings	
*Helicobacter pylori* infection	37 (8.37)
GEP NETs	8 (1.81)
Gastric MALT	1 (0.23)
Early gastric cancer	4 (0.9)

**Table 2:** Comparison of the severity of OLGA/OLGIM depending on selected factors. Abbreviations: see Table 1.

Analyzed Factor		Value					
		OLGA 0–II	OLGA III–IV	*p*-Value	OLGIM 0-II	OLGIM III-IV	*p*-Value
Family history of gastric cancer		147 (42.36)	13 (14.44)	<0.001	153 (39.53)	7 (14)	<0.001
Sex	Male	93 (26.42)	30 (33.33)	0.19	101 (25.77)	22 (44)	<0.007
	Female	259 (73.58)	60 (66.67)		291 (74.23)	28 (56)	
Endoscopy results	Normal	316	64	<0.001	350	30	<0.001
	Suspicious	36	26		42	20	

**Table 3:** Relation between age and severity of OLGA/OLGIM stage, *n* (%). Abbreviations: see Table 1.

OLGA				
Age	OLGA 0–II	OLGA III–IV	N, Total	*p* Value
<45	86 (24.43)	41 (45.56)	127	-
≥45	266 (75.57)	49 (54.44)	315	<0.001
N, total	392	90	442	-
**OLGIM**				
**Age**	**OLGIM 0-II**	**OLGIM III-IV**	**N, Total**	***p* Value**
<45	103 (26.28)	24 (48)	127	-
≥45	289 (73.72)	26 (52)	315	<0.001
N, total	392	50	442	-

**Table 4:** Results of univariate logistic regression assessing the risk of OLGA/OLGIM III-IV stages. Abbreviations: Odds ratio (OR), Confidence interval (CI), see Table 1.

OLGA					
Variable	Variable State	*p* Value	OR	95% CI	
Sex	Female	-	1.000	-	-
	Male	0.19	1.392	0.846	2.292
History of Dysplasia	No	-	1.000	-	-
	Yes	0.17	1.434	0.857	2.399
**OLGIM**					
**Variable**	**Variable State**	***p* Value**	**OR**	**95% CI**	
Sex	Female	-	1.000	-	-
	Male	<0.001	2.264	1.239	4.135
History of Dysplasia	No	-	1.000	-	-
	Yes	0.02	2.087	1.125	3.871

**Table 5:** Results of univariate logistic regression assessing the risk of dysplasia. Abbreviations: see Table 4.

Dysplasia					
Variable	Variable State	*p* Value	OR	95% CI	
Sex	Female	-	1.000	-	-
	Male	0.88	1.036	0.669	1.603
Age	<45	-	1.000	-	-
	≥45	0.17	0.737	0.481	1.130
Family history of gastric cancer	No	-	1.000	-	-
	Yes	<0.001	0.437	0.282	0.677
History of atrophic gastritis	No	-	1.000	-	-
	Yes	<0.001	2.025	1.342	3.055
History of intestinal metaplasia	No	-	1.000	-	-
	Yes	<0.001	2.214	1.476	3.322

## Data Availability

The original contributions presented in this study are included in the article/Supplementary Material. Further inquiries can be directed to the corresponding author.

## Supplementary Materials

The following supporting information can be downloaded at: https://www.mdpi.com/article/10.3390/jcm14196910/s1, Table S1: Results of multivariable logistic regression assessing the risk of OLGA III-IV stages; Table S2: Comparisons of dysplasia presence and endoscopy findings; Table S3: Results of multivariable logistic regression assessing the risk of dysplasia.
