# Supplementary material for: Single-Time Gastroscopy in High-Risk Patients: Screening Effectiveness for Gastric Precancerous Conditions in a Low-To Moderate-Incidence Population
Source: J Clin Med. 2025 Sep 29;14(19):6910. doi: 10.3390/jcm14196910 (PMC12524409; doi:10.3390/jcm14196910)
Supplement: Supplementary file 1 [file jcm-14-06910-s001.zip › jcm-3860006-supplementary.pdf]

Variable		*p*-Value	OR	95% CI	
Sex	Female	-	1.000	-	-
	Male	0.13	1.523	0.885	2.619
Atrophic gastritis	No	-	1.000	-	-
	Yes	<.001	6.470	3.214	13.025
Intestinal metaplasia	No	-	1.000	-	-
	Yes	0.15	1.500	0.860	2.617
Dysplasia	No	-	1.000	-	-
	Yes	0.36	1.300	0.739	2.286

Table S1 Results of multivariable logistic regression assessing the risk of OLGA III-IV stages. Values were considered significant at P <0.05. Abbreviations: Operative Link for Gastritis Assessment (OLGA), Odds ratio (OR), Confidence interval (CI),

	DYSPLASIA			
**Endoscopy result**	**No**	**Yes**	**Total**	***p*-Value**
Normal	258	122	380	
Suspicious	32	30	62	0.01
Total	290	152	442	

Table S2 Comparisons of dysplasia presence and endoscopy findings. Abbreviations: see table S1

Variable		*p*-Value	OR	95% CI	
Sex	Female	-	1.000	-	-
	Male	0.82	1.054	0.667	1.666
Age	≤ 50 lat	-	1.000	-	-
	> 50 lat	0.49	0.853	0.544	1.339
Family history of GC	No	-	1.000	-	-
	Yes	0.18	0.681	0.388	1.195
Atrophic gastritis	No	-	1.000	-	-
	Yes	0.21	1.386	0.833	2.307
Intestinal metaplasia	No	-	1.000	-	-
	Yes	0.02	1.717	1.081	2.729

Table S3 Results of multivariable logistic regression assessing the risk of dysplasia. Abbreviations: see table S1
